# Internal migration, health selection, and the salmon bias: A register-based study of Finland

**DOI:** 10.1016/j.socscimed.2025.118200

**Published:** 2025-05-16

**Authors:** Eugenio Paglino, Irma T. Elo, Pekka Martikainen

**Affiliations:** a Helsinki Institute for Demography and Population Health, Helsinki, Finland; b Max Planck–University of Helsinki Center for Social Inequalities in Population Health, Helsinki, Finland; c Population Studies Center, University of Pennsylvania, Philadelphia, USA; d Max Planck Institute for Demographic Research, Rostock, Germany

**Keywords:** Internal migration, Mortality, Causes of death, Register data

## Abstract

Studies on international migrants have repeatedly found a mortality advantage of migrant over native-born populations. Data artifacts, differential prevalence of health-related behaviors, and health-related selection of immigrants and return migrants have been proposed as explanations. Neither the existence of a migrant mortality advantage for internal migrants nor the validity of existing explanations for this group have been extensively studied. Taking advantage of Finnish register data, we extend the literature on health and internal migration in four ways: 1) by using finer geographic units than previous studies, 2) by adopting models that provide more flexibility compared to alternatives based on the proportional hazard assumption, 3) by distinguishing migrants based on whether they return to their birth region (returnees) or do not (leavers), and by age at migration, and 4) by examining cause-specific mortality. We find that both leavers and returnees enjoy a mortality advantage over non-migrants. For both groups, the mortality advantage relative to non-migrants declines with age but is more pronounced for those who move above age 60 and small or negative for those who move at prime working ages. Circulatory-disease mortality accounts for more than half of the longevity advantage of both leavers and returnees. External and alcohol-related causes also contribute, particularly at younger ages. Our results challenge the idea that findings from studies of international migrants can be fully generalized to internal migrants. We demonstrate a consistent healthy migrant effect for all internal migrants, both those who leave and those who return to their region of birth.

## 1. Introduction

Across high-income countries, international migrants have been found to typically have lower mortality than the native-born populations. This finding, referred to as the Migrant Mortality Advantage (MMA), has been replicated across different settings (Boulogne et al., 2012; Singh and Miller, 2004; Singh and Siahpush, 2001; Wallace et al., 2022; Wallace and Kulu, 2015). Explanations for the MMA include data artifacts (Arias et al., 2010; Palloni and Arias, 2004), health-related selection with respect to both in-migration, the healthy-immigrant effect, and return migration, the salmon-bias effect (Abraído-Lanza et al., 1999; Guillot et al., 2023; Pablos-Méndez, 1994; Turra and Elo, 2008), and health-related behaviors or cultural effects (Blue and Fenelon, 2011; Fenelon, 2013; Riosmena et al., 2017; Singh and Hiatt, 2006).

Hypotheses involving selection of migrants are difficult to empirically assess because they require longitudinal data that follow individuals before they move to the host country, while they reside in the host country, and if they leave the host country. For this reason, direct tests of both the healthy-immigrant hypothesis (Diaz et al., 2018) and the salmon-bias hypothesis (Arenas et al., 2015; Diaz et al., 2016; Guillot et al., 2023; Turra and Elo, 2008) are rare.

Internal migration offers an easier setting in which selection mechanisms can be investigated. Longitudinal surveys or register data can be used to build individual-level internal migration histories following individuals over time as they leave and/or return to their region of birth. This information can be used to compare the health status of those who remain in their region of birth (stayers), those who leave and never return (leavers), and those who leave but eventually come back (returnees). It has also been argued that internal migrants are similar to international migrants in many aspects, including health and earning profiles (Butcher, 1994; Hamilton, 2014, 2015). A comparison of internal migrants with the non-migrant population can thus indirectly inform us about the patterns and drivers of health differences between the immigrant and the native-born population. Evidence from census and survey data suggests that individuals moving within the United Kingdom, Canada, China, Indonesia, Germany, and Italy are healthier than those who remain in their region of birth (Chen, 2011; Debbarman et al., 2023; Lu, 2008; Lu and Qin, 2014; Rasulo et al., 2012; Tong and Piotrowski, 2012; Wallace and Kulu, 2014b; Westphal, 2016). Other studies based on survey data in Brazil (Pescarini et al., 2023), Germany (Holz, 2022) and England and Wales (Green et al., 2015), and on register data in Finland and Sweden (Andersson and Drefahl, 2017; Saarela and Finnäs, 2006, 2008) obtained mixed or opposite results.

In our study, we extend the existing literature by taking advantage of Finnish register data to 1) examine migration flows at the regional level, adding substantially more granularity compared with previous studies, 2) build detailed region-of-residence histories and develop a classification of internal migration statuses distinguishing between stayers, leavers, and returnees, and further classify the latter two groups by age at migration, 3) model mortality differentials between migration groups by using flexible statistical models that allow differences to vary by age, and 4) investigate cause-specific contributions to mortality differentials by migration status.

## 2. Migration, health, and mortality

### 2.1. International migration and the migrant mortality advantage

Studies investigating the relationship between international migration and mortality have documented the presence of a Migrant Mortality Advantage (MMA) across several high-income countries including the United States (Singh and Hiatt, 2006; Singh and Siahpush, 2001), France (Boulogne et al., 2012; Wallace et al., 2019), England and Wales (Wallace and Kulu, 2014a, 2015), the Nordic countries (Kemppainen et al., 2024; Wallace, 2022; Wallace et al., 2022), and Canada (Debbarman et al., 2023; Omariba et al., 2014). Findings from these studies are often deemed paradoxical since immigrants tend to have lower SES than the native-born populations (Markides and Coreil, 1986; Markides and Eschbach, 2005, 2011). Explanations for the lower mortality of immigrant populations generally belong to one of three categories: 1) data artifacts (Arias et al., 2010; Palloni and Arias, 2004; Wallace and Kulu, 2014a), 2) positive health-related selection of migrants, the healthy-immigrant effect (Abraído-Lanza et al., 1999; Diaz et al., 2018; Sorlie et al., 1993), or negative health selection of return migrants, the salmon-bias effect (Guillot et al., 2023; Pablos-Méndez, 1994; Turra and Elo, 2008), and 3) effects of migration including changes in the prevalence of health-related behaviors, such as smoking (Blue and Fenelon, 2011; Fenelon, 2013; Singh and Hiatt, 2006).

The MMA typically exhibits a U-shape pattern over age, with migrants having higher mortality than the native-born population at young ages, a sizeable advantage at adult ages, and mortality convergence at the older ages (Guillot et al., 2018; Hendi and Ho, 2021; Juárez et al., 2018). This pattern is consistent with the healthy-immigrant effect since the selective pressure would be stronger for migrants moving for work or study in early adulthood and less so for migrants moving for family reunification, who typically move at older ages (Guillot et al., 2018). Cultural effects might also produce a U-shape age pattern if migrants experience some degree of acculturation (Guillot et al., 2018), because health behaviors of migrants will converge to those of the native-born population and the degree of protection provided by immigrant social networks will decline with duration of stay (Riosmena et al., 2015).

### 2.2. Internal migration and the migrant mortality advantage

In the context of internal migration, the evidence for the presence of a migrant mortality or health advantage is mixed. Studies in Sweden and Finland (Andersson and Drefahl, 2017; Saarela and Finnäs, 2006, 2008) and one study from Brazil (Pescarini et al., 2023) found no universal mortality advantage among migrants compared to non-migrants in the destination region. One study on the city of Turin in Italy and another one on interprovincial migrants to the Manitoba province in Canada found a mortality advantage for internal migrants (Debbarman et al., 2023; Rasulo et al., 2012). Studies on migrants between England and Wales and Scotland also found a migrants mortality advantage (Popham et al., 2010; Popham and Boyle, 2011). Historical evidence from the city of Rotterdam, the Netherlands is consistent with a mortality advantage of internal migrants (Puschmann et al., 2017), but historical evidence from the United States suggests instead the presence of an internal migrant mortality disadvantage in the early 20th century (Sanchez, 2003). Studies looking at health rather than mortality generally find a health advantage of internal migrants compared to non-migrants in the destination area (Boyle et al., 2002; Norman et al., 2005; Wallace and Kulu, 2014b; Wen et al., 2010). Other studies comparing physical and mental health outcomes between non-migrants and migrants from the same birth region and focusing on adult ages also found more favorable health profiles among migrants compared to non-migrants (Chen, 2011; Lu, 2008; Lu and Qin, 2014; Tong and Piotrowski, 2012; Wallace and Kulu, 2014b; Westphal, 2016).

Few studies have directly investigated the presence of a healthy-immigrant effect by comparing health and mortality of internal migrants with those of non-migrants from the same region of birth. Studies using Swedish and Finnish register data suggest that internal migrants have no mortality advantage over non-migrants from the same region of birth in Sweden (Andersson and Drefahl, 2017) and report mixed finding in Finland (Saarela and Finnäs, 2006, 2008). Two studies from the UK that examined whether internal migration can explain regional differences in health and mortality (Brimblecombe et al., 2000; Norman et al., 2005) find instead evidence that migrants have lower mortality than stayers in their birth region. Another study from the UK finds that migrants from Scotland to England have better health than non-migrants from Scotland, but this finding does not extend to migrants from England to Scotland (Wallace and Kulu, 2014b). Regarding health-selection of return migrants, Andersson and Drefahl (2017), find strong evidence of negative selection, with return migrants having significantly higher mortality than migrants who do not return. Lu and Qin (2014), who look at whether self-reported health predicts return migration among internal migrants, similarly find that healthier individuals are less likely to return. These finding are consistent with the presence of a salmon-bias effect as discussed in the international migration literature. However, Wallace and Kulu (2014b), who look at migration between England and Scotland, do not find evidence of a Salmon bias in the prevalence of long-term illness.

### 2.3. The Finnish context

In Finland, mortality generally increases when moving from the Southwest to the Northeast (Saarela and Finnäs, 2006). The Southwest to Northeast mortality divide extends to region of birth (Elo et al., 2014), even when controlling for current region of residence (Saarela and Finnäs, 2005, 2011). Furthermore, geographic differences in mortality have not declined in the past 40 years (Suulamo et al., 2021; Wilson et al., 2020).

Internal migration occurs predominantly from Northeast to Southwest regions, thus from high-mortality to low-mortality areas (Kupiszewski et al., 2000). After World War II and following rapid industrialization of the economy, Finland saw a progressive concentration of the population in urban areas with substantial rural to urban migration, driven by demand for labor in urban centers of the Southwest (Kultalahti, 2001; Kupiszewski et al., 2000). One study of reasons for migration among internal migrants in the late 1970s found that work was the main reason for internal migration, particularly for those moving from rural to urban areas (Nieminen, 1983). While internal migration slowed down in the 1980s, it experienced a new resurgence in the 1990s, with the rapid growth of the high-tech sector attracting high-skilled workers to urban areas (Kultalahti, 2001). The combined presence of regional variation in mortality and large migration flows from high-mortality to low-mortality areas, in addition to the availability of full population data, makes Finland an ideal setting to investigate the relationship between internal migration and mortality.

### 2.4. Summary of existing gaps and our contributions

All the studies we reviewed on internal migration and mortality summarize the mortality differences between migrants and non-migrants using a single indicator and do not examine if and how the migrant mortality advantage varies over age. Additionally, existing studies classify potentially complex migration histories into a limited number of categories. Saarela and Finnäs (2006) take the point of view of the receiving region and compare non-migrants born in the region (reference) to in-migrants from any other region and to out-migrants from that region who moved to any other region. This design prevents the authors from drawing any conclusions about the mortality of return migrants who are combined with non-migrants. The same is true for Popham and Boyle (2011) and Popham et al. (2010). The study using data from the Turin Longitudinal Study (Rasulo et al., 2012) and the one using Canadian data on Manitoba province (Debbarman et al., 2023), only compare in-migrants with individuals born in the destination region. Similarly, the study on internal migration in Brazil (Pescarini et al., 2023) compares internal migrants everywhere with non-migrants, confounding the healthy-immigrant effect, the salmon-bias effect, and other mechanisms that may give rise to the migrant mortality advantage. The design of Andersson and Drefahl (2017) is ideal to investigate selection effects, but their focus on a single migration flow (between Northern and Southern Sweden) means that within-region differences in the distribution of migrants, both before they moved and after they returned, could bias the results if there is within-region variation in mortality. Finally, all studies focus exclusively on all-cause mortality. While this is an important indicator, cause-specific mortality has the advantage of revealing more information about potential underlying mechanisms.

In comparison to international migration, research evidence on the mortality of internal migrant and return-migrants is thus limited and results remain inconclusive. Using unique Finnish register data covering the total population, we propose to fill this gap and alleviate the limitations of existing studies in four ways. First, by using finer geographic units compared to previous studies, the 21 NUTS 3 regions of Finland, we will avoid possible biases rising from heterogenous migration experiences captured by large area units. Second, by adopting models that provide more flexibility compared to alternatives based on the restrictive proportional hazard assumption, thus allowing us to capture age-specific effects and calculate life expectancies and other indicators of longevity differentials. Third, by distinguishing migrants based on age at first migration out of the region of birth and age at return, for those eventually returning, allowing us to investigate how both selective migration and the salmon-bias effect depend on age at migration. Finally, by complementing all-cause with cause-specific mortality analyses, we will gain important insight into the mechanisms driving the observed mortality differentials.

## 3. Data and methods

### 3.1. Sample

We use individual-level data for the total Finnish population from 1970 to 2018. We combine data from the 1970, 1975, 1980, and 1985 censuses with annual data from administrative registers for the period 1987–2018. Socioeconomic and demographic data from censuses and administrative registers were linked by Statistics Finland to death records using personal identification numbers. The linkage rate is above 99 % (Statistics Finland, 2023). We include every individual born in Finland, excluding the part of Karelia that was ceded to the Soviet Union after World War II, who was alive in 1970 and did not leave Finland between 1970 and 2018 and follow them until death or the end of the follow-up in December 2018. We consider deaths and person-years of exposures from age 20 to age 95. The ages included in this study account for approximately 95 % of all deaths. Table 1 summarizes the characteristics of the sample (see the Supplementary Material for more details).

### 3.2. Building and classifying migration histories

We compute individual person years of exposure by calendar year and single years of age and then aggregate person-years of exposure and death counts by calendar year, three-year age groups, sex, region of birth, and a categorical variable capturing migration status. Moves are identified by looking at whether the region of residence on December 31st changed from one year to the next. We only consider moves between NUTS 3 region and not within regions (see Fig. S1 for a map of NUTS 3 regions). We differentiate between individuals who never left their region of birth, “stayers”, individuals who left their region of birth and did not return, “leavers”, and individuals who left their region of birth but returned before dying, “returnees”. Some individuals will reside in multiple regions over their lives. To simplify the analysis, we consider as stayers only individuals who were never observed outside their region of birth. Leavers include all individuals who left their region of birth at some point and were still residing in a different region at the end of follow-up or in the year of death. Finally, returnees include all individuals who left their region of birth at some point but were again residing in their region of birth on December 31st, 2018, or in the year of death. We further disaggregate leavers by age at migration (before age 17, between ages 17 and 29, between ages 30 and 59, and after age 59), and returnees by the age at which they return to their region of birth (before age 30, between ages 30 and 59, after age 59). We use the term “movers” to jointly refer to leavers and returnees.

### 3.3. Addressing censoring of age at migration

Residential histories for some individuals in our population are subject to left censoring, interval censoring, or both. Left censoring arises because while birth region is known for every individual, we only observe current region of residence starting in 1970. Approximately 18 % of the sample is affected by left censoring of age at migration. Interval censoring arises for residential histories prior to 1986 because for the 1970–1986 period we observe individuals only in the 1970, 1975, 1980, and 1985 censuses. Thus, when a change of residence is observed before 1987, it cannot be precisely placed in time. We treat this situation as a missing data problem and use models for censored data (Anderson-Bergman, 2017) to impute age at migration for affected individuals. Further details are provided in the Supplementary Materials.

### 3.4. Causes of death

Cause-specific analyses can provide substantially more information than those relying on all-cause mortality alone, particularly regarding mechanisms underlying mortality disparities. For this reason, we further classify deaths by selected groups of causes. We distinguish between neoplasms (ICD10 codes C00-D48), dementia and Alzheimer’s disease (F01, F03, G30, R54), diseases of the circulatory system (I00-I425, I427-I99), diseases of the respiratory system (J00-J64, J66-J99), all alcohol-related diseases, which are excluded from all other categories, and external causes (V01-X44, X46-Y89, U129). Deaths with unknown cause or ill-defined causes (2.1 % of all deaths) were assigned proportionally to the other categories stratifying by 5-year periods, 5-year age groups, and sex.

### 3.5. Statistical models

We estimate Negative-Binomial Generalized Additive Models (GAM) to assess the relationship between age-specific mortality and migration status while adjusting for region of birth and calendar year. Poisson or Negative Binomial models are widely used in mortality modeling (Alexander et al., 2017; Bryant and Zhang, 2020). Relative to Poisson models, Negative Binomial models should be preferred when overdispersion may be of concern. Compared with semi-parametric approaches, such as Cox models, Negative Binomial models allow us to directly compute different measures of longevity, including life expectancies, differences in life expectancy, and mortality rate ratios at different ages. At the same time, the use of a GAM framework greatly relaxes functional-form assumptions. We perform separate analyses by sex and cause of death. We denote with $D_{x,t,r,m}$ the number of deaths occurred in year t among those aged x to x+2, born in region r, and with migration status m. The corresponding exposures are denoted with $E_{x,t,r,m}$, and the corresponding mortality rates with $M_{x,t,r,m}$. Our model is then:

$$D_{x,t,r,m}\sim \text{NegativeBinomial}(m_{x,t,r,m}\cdot E_{x,t,r,m},\theta )\;\text{log}(m_{x,t,r,m})=\beta_{r}+\beta_{t}+\beta_{m}+s_{m}(x)$$

Where $m_{x,t,r,m}$ is the mean of the Negative Binomial distribution and θ is its overdispersion parameter. The model includes coefficients for birth region ($\beta_{r}$), calendar year ($\beta_{t}$), and migration status ($\beta_{m}$). Age is modeled with a smooth function $s_{m}(\cdot )$, which is allowed to differ by migration status. We set x to be the mid-point of each 3-year age interval. The smooth functions, defined as linear combinations of 10 cubic splines with penalized coefficients, are expressed as deviations from the status-specific intercept $\beta_{m}$ and constrained to sum to zero for identifiability. Models are fit with the bam function from the mgcv package (Wood, 2023) for the R programming language.

To handle the fact that some causes of death have low or no mortality at some ages, we limit the age ranges on which some of the cause-specific models are fit by keeping only ages in which at least 0.5 % of all deaths from a given cause occurred or there were at least 1000 deaths across all years.

Regarding migration status, in order to observe age at out-migration or age at return migration above a given age, the individuals moving need to have survived to that age. For example, all individuals in the group “Returnees after age 59” will have survived to age 59. For these groups, mortality before the lower end of their age-at-migration interval is zero by definition and is not modeled. Additionally, for the group “Returnees before age 30” we do not observe any deaths above age 77 and thus we set this age as the upper limit for this group. For someone to fall into this category they must have been observed at least once in a region different from their birth region and subsequently again in their region of birth, which means that the oldest individuals in this group will be 29 in 1970 and 77 in 2018.

### 3.6. Computing expected years lived and decomposing differences by age and cause

Using estimated mortality rates for stayers, leavers, and returnees, we employ standard techniques (Preston et al., 2001) to construct group-specific partial life tables for ages 20–95. We then use these life tables to compute differences in expected number of years lived between selected ages and age 95 (i.e., temporary life expectancies) between leavers and returnees by age at migration and stayers, as the reference group. We then decompose these differences by age and cause of death using the line-integral decomposition (Horiuchi et al., 2008).

## 4. Results

### 4.1. All-cause mortality of stayers, leavers, and returnees

**Fig. 1.**
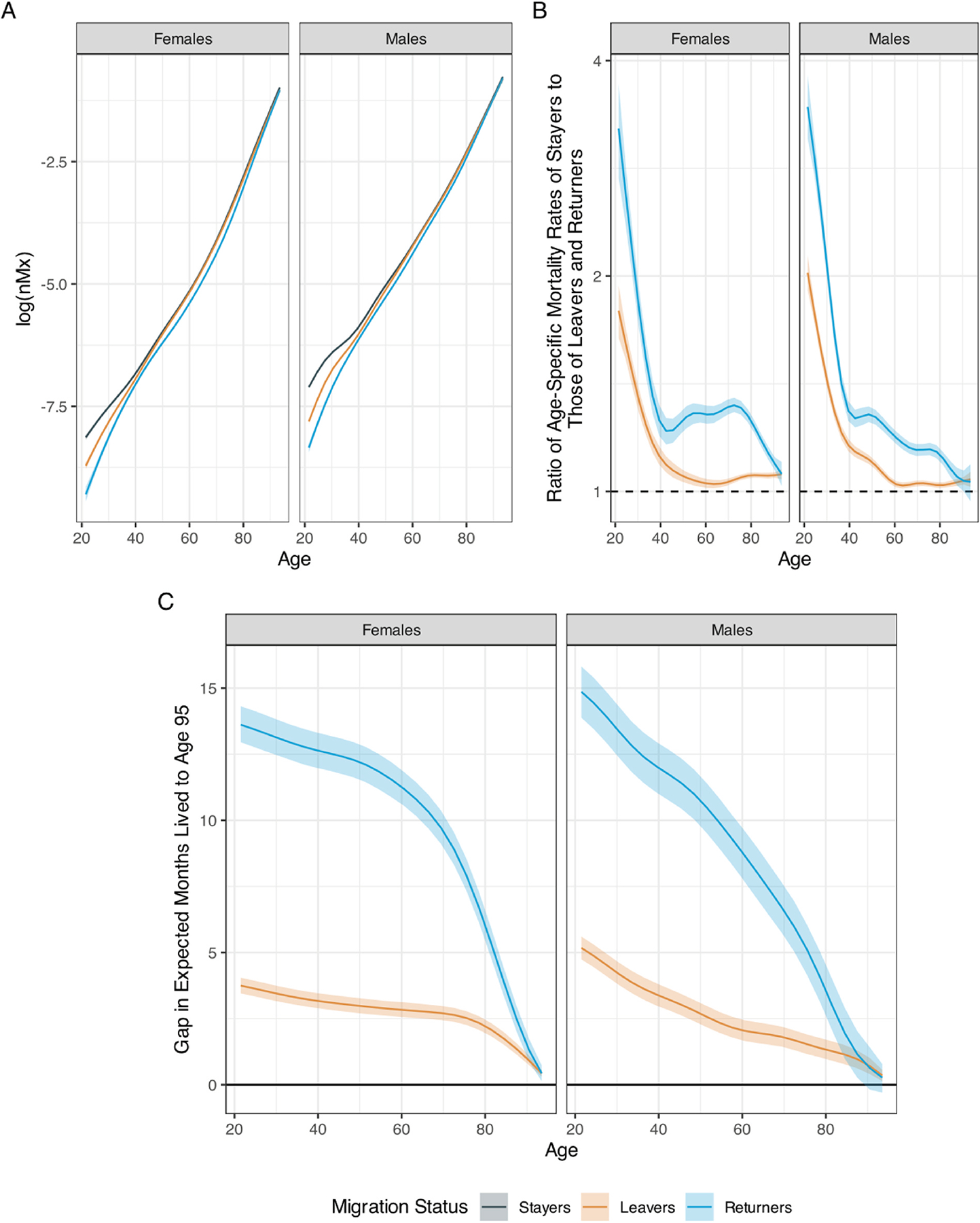
Age-Specific log Mortality Rates (Panel A), Age-Specific Mortality Rate Ratios (Panel B), and Gap in Years Lived to Age 95 (Panel C) by Sex and Migration Status. Notes: For both Panel B and Panel C, stayers are the reference category. Mortality rates for stayers are thus in the numerator of the rate ratios and the gap in years lived to age 95 in Panel C represents a life expectancy advantage of leavers and returnees with respect to stayers. The shaded areas in panels A and B represent 90 % confidence intervals.

Fig. 1A shows the estimated age-specific mortality rates of stayers, leavers, and returnees. The rates are obtained by conditioning on all combinations of region of birth and year of death and then averaging, as in the usual procedure to obtain average marginal effects. All estimates that follow are presented in the format mean [5th Percentile, 95th Percentile].

We observe a sizeable mortality advantage for both leavers and returnees compared with stayers, for both sexes. The advantage is largest at the younger ages and declines throughout working and older ages. While the mortality of leavers eventually converges to that of stayers, returnees maintain a mortality advantage even at older ages. Fig. 1B displays mortality rate ratios with age-specific mortality rates of stayers in the numerator and those of leavers and returnees in the denominator. Ratios above 1 indicate higher mortality for stayers. Stayers have significantly higher mortality at age 21.5 than both leavers (male ratio: 2.02 [1.91, 2.14], female ratio: 1.79 [1.64, 1.94]) and returnees (males: 3.45 [3.11, 3.82], females: 3.21 [2.74, 3.71]). The advantage declines rather sharply for both groups until age 39.5 (male leavers: 1.16 [1.14, 1.18], female leavers: 1.12 [1.09, 1.16], male returnees 1.30 [1.25, 1.34], female returnees: 1.26 [1.19, 1.32]). For females, the advantage for leavers continues to decline between age 40 and age 60 and virtually disappears at age 60.5 (1.03 [1.01, 1.04]) with a slight increase at older ages. Returnees maintain an advantage which only begins to decline after age 75 (at age 75.5: 1.31 [1.28, 1.34]). For males, the advantage among leavers declines from age 40 to age 60 (at age 60.5: 1.02 [1.01, 1.03]) and slightly increases thereafter. Returnees in contrast maintain a more stable and sizeable advantage until about age 75 (at age 75.5: 1.15 [1.12, 1.17]), with a more marked decline only seen thereafter (see also Table S1).

As can be seen in Fig. 1C, the overall mortality advantage of leavers translates into 5.2 additional months of male life expectancy at age 20 (a 0.7 % difference compared with the temporary life expectancy of stayers), which reduces to 2.6 additional months at age 50 (0.6 %), and 1.3 months at age 80 (1.2 %). The corresponding figures for females are 3.7 months (0.5 %) at age 20, 3.0 months (0.7 %) at age 50, and 2.1 months (1.9 %) at age 80. Due to their more pronounced mortality advantage, the figures are higher for returnees. The additional male life expectancy for returnees is 1 year and 3 months (2.0 %) at age 20, 10.5 months (2.6 %) at age 50, and 3.0 months (2.7 %) at age 80. The corresponding figures for females are 1 year and 2 months (1.8 %) at age 20, 1 year (3.0 %) at age 50, and 5.3 months (4.8 %) at age 80 (see also Table S2).

### 4.2. Cause-specific mortality of stayers, leavers, and returnees

**Fig. 2.**
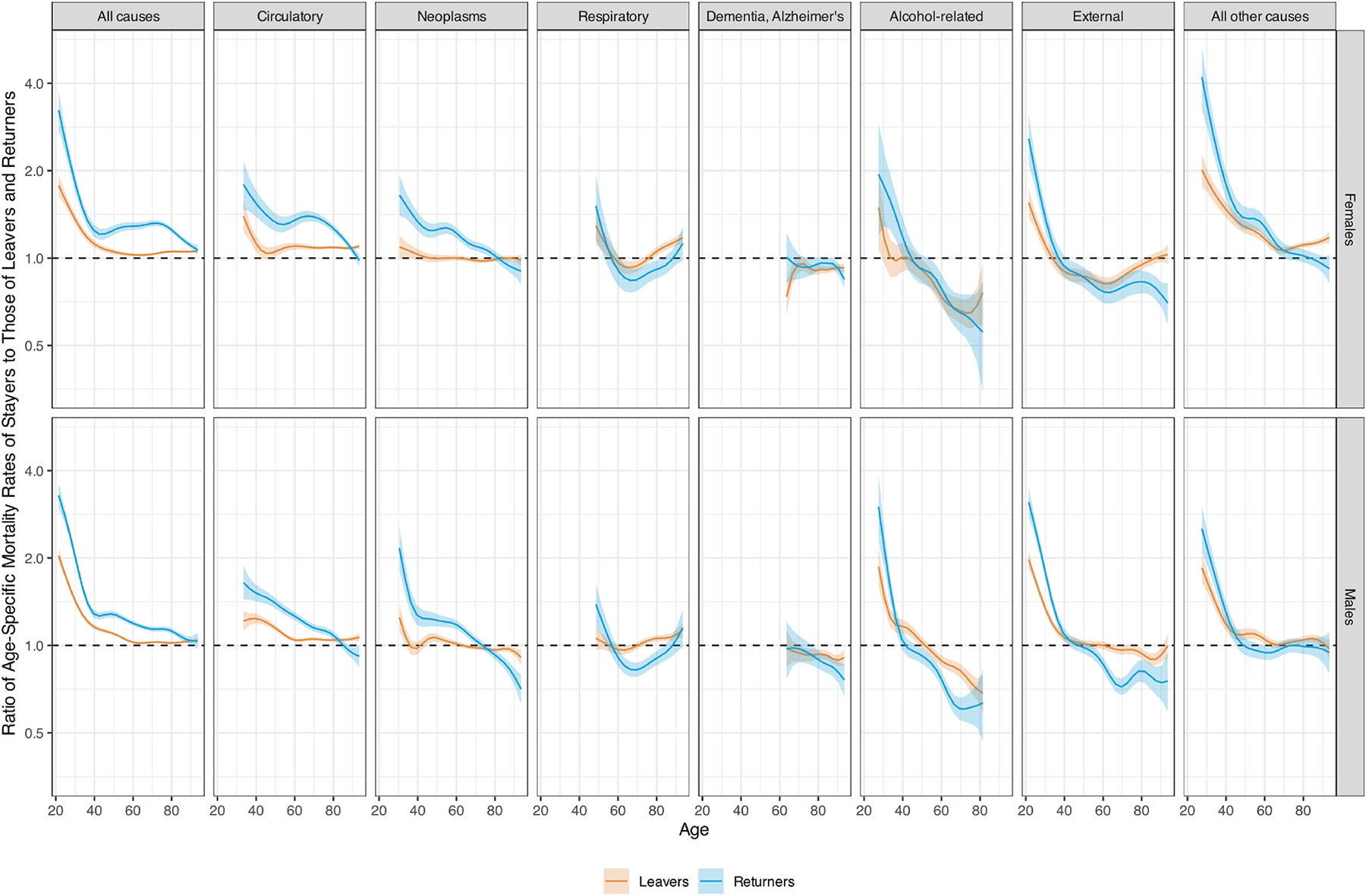
Cause-Specific Mortality Rate Ratios by Sex and Migration Status. Notes: The shaded represent 90 % confidence intervals.

Before age 40, both leavers and returnees have lower mortality from circulatory diseases, alcohol-related, and external causes (Fig. 2). Returnees also have lower neoplasms mortality than stayers. Between age 40 and age 70, returnees’ advantage for all these causes declines and turns into a disadvantage for alcohol-related and external deaths. Above age 70, the returnees’ advantage in circulatory, neoplasms, external, and alcohol-related mortality turns into a disadvantage. Leavers maintain an advantage in circulatory mortality, have no disadvantage in mortality from external causes, but have a disadvantage in alcohol-related mortality. Mortality from respiratory causes is lower among returnees and leavers than stayers at age 45. This advantage declines and turns into a disadvantage for both groups before increasing again after age 65. Only leavers enjoy a respiratory mortality advantage after age 80. Mortality from dementia and Alzheimer’s is higher among both leavers and returnees than stayers, particularly after age 80.

**Fig. 3.**
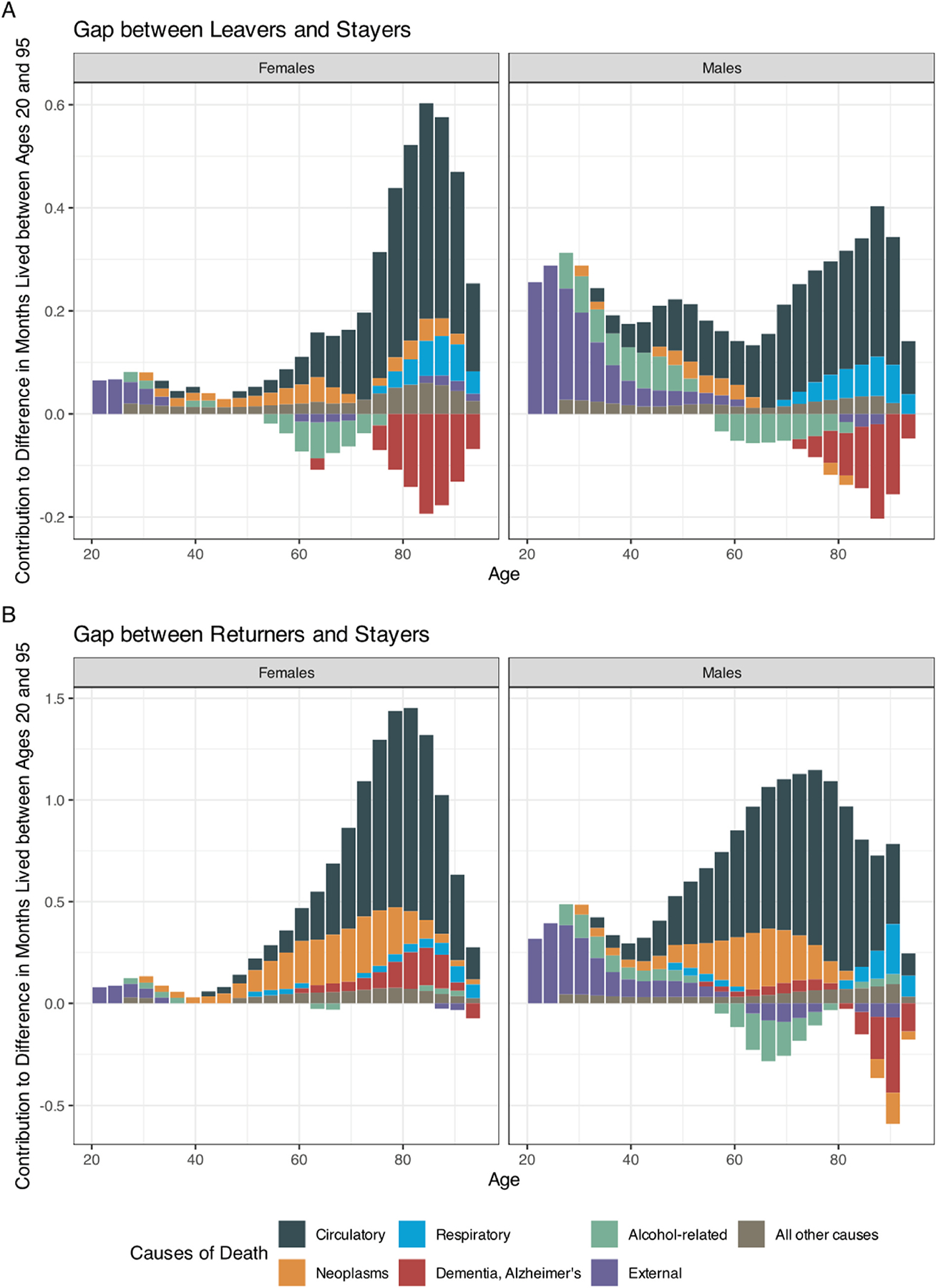
Cause-Specific Contributions to the Gap in Years Lived between Age 20 and Age 95 between Leavers and Stayers (Panel A), and Returnees and Stayers (Panel B). Notes: Decomposition results obtained by using the line-integral decomposition method. Bars in each subplot sum to the total difference in years lived to age 95 between the two groups (e.g. gap between female leavers and female stayers in the top-left panel).

External and alcohol-related causes make a large positive contribution to both the leavers’ (Fig. 3A) and the returnees’ (Fig. 3B) advantage before age 50, particularly for males. Alcohol-related mortality has a positive contribution until age 40, but a negative contribution thereafter, except for female returnees. Overall, external and alcohol-related mortality accounts for 30.2 % of the leavers’ longevity advantage among males but has almost no contribution among females. The corresponding figures for returnees are 10.1 % (males) and 3.0 % (females). The contribution of circulatory diseases is positive at all ages and accounts for more than half of the longevity advantage for both leavers and returnees and both sexes. Mortality from neoplasms has a relatively modest contribution to the leavers’ advantage (14.4 % for females and 4.0 % for males) but a substantial one to the returnees’ advantage (21.9 % for females and 15.0 % for males). For all groups except female returnees, mortality from dementia and Alzheimer’s diseases contributes negatively to the mortality advantage. Finally, respiratory diseases have a residual role, positive but confined to the older ages (see also Table S3 and Table S4).

### 4.3. Age at first migration, age at return migration, and the migrant mortality advantage

**Fig. 4.**
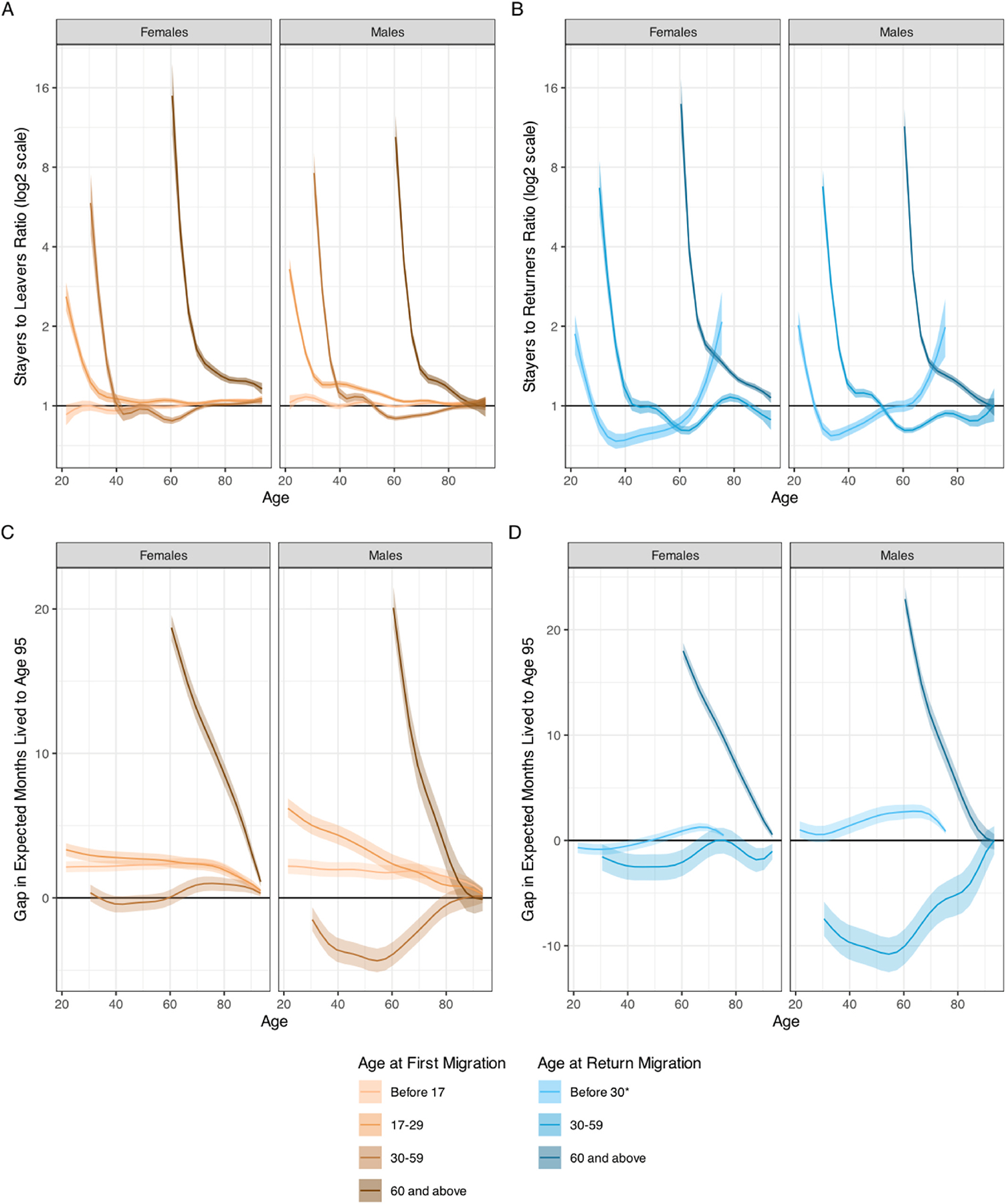
Age-Specific Mortality Rate Ratios for Leavers (Panel A) and Returnees (Panel B), and Gap in Years Lived to Age 95 between Leavers and Stayers (Panel C) and between Returnees and Stayers (Panel D), by Sex and Age at Migration. Notes: In all panels, stayers are the reference category. Mortality rates for stayers are thus in the numerator of the rate ratios in Panel A and B, and the gap in months lived between ages 20 and 95 in Panel C and D represents a life expectancy advantage of leavers and returnees with respect to stayers. The shaded areas in panels A and B represent 90 % confidence intervals. *For returnees before age 30, the differences are calculated for years lived between ages 20 and 77.

For both leavers (Fig. 4A) and returnees (Fig. 4B), and for every age-at-migration group, the relative mortality advantage with respect to stayers is highest at the first age in the age-at-migration interval. At the same time, for both leavers and returnees, the relative advantage is highest for those who move at age 60 or above. Leavers and returnees who move at younger ages, despite generally starting from a large relative advantage, see their advantage quickly disappear and, in the case of returnees, turn into a disadvantage before their mortality rates once again approach those of stayers at the older ages.

The gaps in years lived to age 95 in Fig. 4C, for leavers, and Fig. 4D, for returnees, offer a concise summary of these patterns (see also Table S5). Leavers who move before age 30 and those that move after age 59 enjoy a life expectancy advantage. This longevity advantage amounts to 2.2 months (0.3 % of stayers’ temporary life expectancy) at age 20 for male leavers who move before age 17, 6.2 months (0.8 %) at age 20 for male leavers who move between ages 17 and 29, and 1 year (4.8 %) at age 65 for male leavers who move after age 59. The corresponding figures for females are 2.1 months (0.3 %), 3.3 months (0.4 %), and 1 year and 3 months (6.0 %). Female leavers who move between ages 30 and 59 have practically no advantage relative to stayers, while males have a small disadvantage. For returnees, the survival advantage is limited to males who return before age 30 and everyone who return at ages 60 and above. For males returning before age 30, the advantage in years lived to age 77 is 2.4 months (0.8 %) at age 50 and 2.8 months (2.1 %) at age 65. The advantage for those returning at ages 60 and above is 1 year and 3 months for males at age 65 (6.0 %) and 1 year 2 months for females (5.8 %). Females returning before age 60 have no survival advantage and males returning between ages 30 and 59 have a rather large disadvantage, 9.1 months at age 35 (1.6 %), which grows to 10.7 months at age 50 (2.6 %).

## 5. Discussion

In this study we investigate mortality differentials between internal migrants and non-migrants at adult and older ages. The motivation for our analysis is twofold. First, we want to understand whether empirical findings regarding mortality differentials between international migrants and non-migrants residing in the origin and destination countries extend to internal migrants. Second, we wish to overcome selected methodological and conceptual limitations of the existing literature on internal migration and mortality, including the use of broad geographic areas to classify individuals as internal migrants, the lack of distinction between migrants who eventually return to their birth region (returnees) and those who do not (leavers), the use of models assuming constant differences between groups over age, and the lack of cause-specific analyses. Finland offers an optimal case for this study because of relatively large regional mortality differentials (Saarela and Finnäs, 2006), substantial internal migration, and availability of register data with the possibility of constructing long-term residential histories.

We find that both leavers and returnees enjoy a mortality advantage over non-migrants which is highest at young adult ages and declines thereafter. The lower mortality of leavers translates into 6 additional months of life expectancy between age 20 and age 95 for males and 4 months for females. The advantage is larger for returnees, 1 year and 4 months for males and 1 year and 2 months for females. These advantages are not trivial but are of smaller magnitude compared, for example, with the life expectancy advantage of the Hispanic population in the United States vis-à-vis the White population between the same ages – 25.2 months for males and 31.2 for females in 2019 (Arias and Xu, 2022). For leavers, the mortality advantage is more pronounced for those who leave later in life and is small (females) or absent (males) for those leaving at prime working ages (30–59). The pattern is similar for returnees, with a large advantage among those returning to their birth region after age 60, possibly following retirement, and no advantage, females, or a disadvantage, males, for those returning between ages 30 and 59. Circulatory-disease mortality is responsible for more than half of the longevity advantage of both leavers and returnees. Lower mortality from external and alcohol-related causes is also important, especially for younger males. Dementia and Alzheimer’s are the only causes of death with an advantage for stayers.

Our results differ somewhat from previous studies of mortality and internal migration with a similar design (Andersson and Drefahl, 2017; Saarela and Finnäs, 2006, 2008). First, we find strong evidence that leavers have lower mortality than stayers. This mortality advantage is consistent with the presence of positive selection on health and health-related variables. Second, and in contrast with Andersson and Drefahl (2017), we find that returnees have lower mortality than both stayers and leavers from the same birth region (Fig. S2). This finding runs counter the salmon-bias hypothesis. Our results have a precedent in those of Wallace and Kulu (2014b) for England-Scotland migration, although the study design is quite different. One crucial difference with previous studies is the age range examined. Although the relative mortality advantage of movers is highest at young ages, those moving at ages 60 or above have the largest relative advantage for both leavers and returnees. These two groups of individuals were excluded from some previous studies focusing exclusively on migration at working ages – 16–53 for Andersson and Drefahl (2017) and 40–59 for Saarela and Finnäs (2008). Saarela and Finnäs (2006) include individuals moving at older ages (60–94), but their study differs from ours in two key aspects. First, they combine stayers and returnees in a single category and compare them with leavers and with in-migrants from other regions. If returnees have lower mortality than both stayers and leavers, comparing leavers to a combination of stayers and returnees would reduce the leavers’ advantage. Second, the authors present estimated rate ratios adjusting for educational attainment and family type. If the lower mortality of leavers is related to their higher educational attainment and to differences in family type, adjusting for these variables would make our estimates and those of Saarela and Finnäs (2006) hard to compare.

We find support for extending the healthy-immigrant hypothesis to internal migration, confirming findings from previous studies. We were unable to investigate directly whether the advantage of leavers and returnees vis-à-vis stayers is a product of higher socioeconomic status or other individual level characteristics of the movers. However, the contrast between our findings and those of Saarela and Finnäs (2006) suggests that differences in educational attainment and family type might play a role. In fact, Saarela and Finnäs (2008) find that controlling for educational attainment decreases the mortality advantage of internal migrants, reflecting the better education of movers than stayers. The fact that external and alcohol-related deaths, both displaying marked socioeconomic gradients in Finland (Herttua et al., 2007; Saarela and Finnäs, 2008; Tarkiainen et al., 2016), are important contributors to the mortality advantage of male leavers and returnees offers additional evidence that higher SES is one of the mechanisms driving our results. These findings stand in contrast with the large literature on the Hispanic Mortality Paradox in the United States (Markides and Coreil, 1986; Markides and Eschbach, 2005, 2011), which states that the Hispanic population in the United States, with a large proportion foreign-born, enjoys a mortality advantage vis-à-vis the non-Hispanic White population despite having lower socioeconomic status.

In contrast with the salmon-bias hypothesis in the international migration literature, we find no evidence that returnees have higher mortality than either leavers or stayers. Instead, returnees are the group with the lowest mortality. Theoretical reasons for negative health selection of international return migrants include the desire to die in their country of origin (Razum et al., 2005; Tezcan, 2019), seeking family support and more affordable health care (Diaz et al., 2016), and health deterioration potentially changing the economic incentives to remain in the host country (Guillot et al., 2023). A priori it is hard to know whether these mechanisms would also apply to internal migration. For example, a preference for dying in the country of origin might not extend to region of birth and healthcare costs might be similar across regions (Wallace and Kulu, 2014b). Economic incentives to remigrate might also differ between international and internal migrants. While economic opportunities for international migrants might be dramatically different in the host and origin country, this may be less likely the case or of lesser magnitude for internal migrants moving between regions. Our finding that return migrants moving back relatively early in adulthood have a mortality disadvantage, while those moving close to or after retirement enjoy an advantage, is consistent with neoclassic theories of migration (Harris and Todaro, 1970; Massey et al., 1993; Todaro, 1969). Younger individuals in prime working ages could be facing higher living costs in their destination region, which could exceed the advantage from added economic opportunities if poor health diminishes the migrants’ labor market chances. Older individuals, whose earnings may come in large part from pension payments, may face the opposite incentives if they had moved to regions with higher labor income but also higher costs of living. Returning after retirement may thus make economic sense for retirees, but health issues might prevent a negatively selected subgroup from moving back to their birth region.

### 5.1. Methodological considerations

The data used in our study come with advantages, including large samples, consistent measurement of region of birth and region of residence, and no loss to follow-up. However, the paper also comes with certain limitations. First, left censoring affects our ability to observe age at first migration and age at return migration for those born before 1970. We mitigated this problem by imputing these two variables based on models for censored data. In sensitivity analyses, we found that limiting the analysis to cohorts born in or after 1970 did not substantially change the results (see the Supplementary Material). Second, the focus of the study and the use of aggregate data mean we do not examine how individual-level characteristics explain differences among leavers, returnees, and stayers. Future research should examine these factors including time spent in the destination region, distance between birth and destination regions, motivations for migration, as well as direct assessment of health status before and after each migratory event. An additional direction for future research is to investigate how health and mortality of migrants vary depending on whether individuals are moving from areas with lower socioeconomic status to areas with higher socioeconomic status or vice versa.

## 6. Conclusions

In this study we document a sizeable advantage of internal migrants with respect to non-migrants from the same region of birth in Finland. We additionally find that those who eventually return to their region of birth enjoy an additional longevity advantage, making them the group with the lowest mortality. Our findings contribute to the literature on internal migration and mortality. Of the three sets of substantive explanations for the migrant mortality advantage, we find support for positive health-related selection of movers, possibly driven by health-related behaviors and higher SES of movers, but no support for higher mortality among migrants who return to their region of birth, i.e., salmon-bias effect. Thus, simply extending existing theories of migration and health formulated based on international migration to internal migrants is likely to be flawed. A re-examination of factors driving internal migration and their relationships with health and health-related variables will help inform theories at the intersection of internal migration, health, and mortality. Beyond their importance for scholarly understandings of health and migration, our results are relevant for policymakers wishing to understand how internal migration flows may affect regional demand for healthcare services and inform them and researchers on how internal migration contributes to regional differences in mortality.

## Supplementary Material

Appendix A

Appendix A. Supplementary data

Supplementary data to this article can be found online at https://doi.org/10.1016/j.socscimed.2025.118200.

## Figures and Tables

**Table 1:** Descriptive characteristics of the sample.

Sample Characteristics (N = 5,751,446)	Categorical: n (%)
	Continuous: Mean (SD) [Min, Q1, Q2, Q3, Max]
Sex	
Females	2,902,769 (50 %)
Males	2,848,677 (50 %)
Age in 2018 (for survivors)	51 (18) [20, 36, 52, 66, 106]
Year of Birth	1951 (28) [1867, 1930, 1953, 1974, 1998]
Dead	2,006,604 (35 %)
Age at Death	72 (15) [20, 64, 75, 83, 112]
Year of Death	1995 (14) [1971, 1984, 1996, 2008, 2018]
Age at migration is left censored	1,042,405 (18 %)
Age at First Migration	19 (13) [0, 8, 20, 25, 101]
Age at Return Migration	34 (16) [1, 24, 30, 42, 101]
Number of Observations	27 (12) [1, 22, 33, 36, 36]
Number of Moves	1 (1) [0, 0, 0, 1, 21]
Migration Status	
Leaver (<17)	861,788 (15 %)
Leaver (17–29)	862,263 (15 %)
Leaver (30–59)	228,160 (4.0 %)
Leaver (60 and above)	27,781 (0.5 %)
Returnee (<17)	28,138 (0.5 %)
Returnee (17–29)	220,391 (3.8 %)
Returnee (30–59)	199,511 (3.5 %)
Returnee (60 and above)	50,946 (0.9 %)
Stayer	3,272,468 (57 %)

Notes: For categorical variables we report the number of individuals in each category followed by the proportion of the total in parentheses. In summary, the statistics for categorical variables follow the format: n (%). For continuous variables we report the average followed by the standard deviation, in round parentheses, and the minimum, 1st quartile, median, 3rd quartile, and maximum, in square parentheses. In summary the statistics for categorical variables follow the format: Mean (SD) [Min, Q1, Q2, Q3, Max].

## Data Availability

The authors have made R codes used to analyze the data available in an online repository at https://osf.io/jzy2p. Due to data protection regulations of the national register-holders providing the data, the authors are not allowed to make the data available to third parties. Interested parties have the possibility of gaining data acess by contacting Statistics Finland.
